# Medicinal plant-based health products: Where is the medicinal constituent?

**DOI:** 10.4103/0253-7613.56063

**Published:** 2009-08

**Authors:** R. Mathur, T. Velpandian

**Affiliations:** Departments of Pharmacology and Ocular Pharmacology, Delhi Institute of Pharmaceutical Sciences and Research, New Delhi, India. E-mail: mathurajani@yahoo.com; 1 Dr. Rajendra Prasad Centre for Ophthalmic Sciences, All India Institute of Medical Sciences, New Delhi, India

Sir,

Ancient literatures like Chinese, Unani, Ayurveda, and Siddha extol the virtues of plant-derived medicines. Their preventive and therapeutic benefits have been defined for almost all ailments. The long-standing use of herbal medicines and the belief that they are safe and inexpensive have collectively contributed to their mass appeal. The World Health Organization (WHO) estimates that about 80% of the population in developing countries relies on herbal medicines at least for their primary healthcare and an equal proportion in developed countries have tried this approach at least once.[1] However, there are increasing number of reports in the literature that are questioning the quality of herbal medicines and it is feared that the public is being exposed to a major health risk due to misidentification, adulteration, and contamination of these “health products.”

This study was conducted with the aim to bring out the facts by randomly selecting medicinal plant-based products that are available in the market and assess their quality. The products of Ashwagandha (Withania somnifera, Apocynaceae) were chosen, as it is one of the most popular and commonly used medicinal plants. The steroidal lactones (withanolides) present in its roots have been implicated with a wide range of therapeutic activities like anti-cancer, anti-epileptic, memory enhancer, mood elevator, rejuvenator, stress reliever, anti-ageing, anti-oxidant, and in general adaptogenic effect. They have also been identified as “marker compounds” for chemical standardization of Ashwagandha-based products. In this study, a simple high-performance liquid chromatography based method was developed to quantify the withanolide and withaferin in different commercially available Ashwagandha-based products.

The four commercial samples of Ashwagandha were in the form of tablets (samples A and B), powder (sample C), and syrup (sample D). The film coating of the tablets was carefully scraped off and three tablets were triturated. The 1 g of tablet powder was refluxed (methanol, 100 ml), and concentrated to give a methanolic extract. Similarly, the methanolic extract of the powder sample C (1 g) was prepared. Sample D (5 ml) was first lyophilized to give a dry powder, which was extracted using methanol, by the aforementioned method.

Standard withanolide-A (10 μg/ml) and withaferin-A (20 μg/ ml) were prepared and used to set the standard curve. The Nova-pak^®^ column (RP-C18, 4 mm,150 × 30 mm i.d.; Waters, Milford, USA) was used as the stationary phase. A freshly filtered mobile phase (potassium-dihydrogen orthophosphate (0.05 M): acetonitrile:3:2, v/v) was used to isocratically elute samples (20 μl) at the flow rate of 1 ml/min for a run time of 25 minutes. The samples were run in triplicate under constant PDA detection, and analytical wavelength was set at 229 nm. All calibration curves of withanolide-A and withaferin-A were made with correlation values of at least 0.995. The inter-day variabilities for withanolide-A and withaferin-A were assayed at concentrations of 0.062-2.0 μg/ml on three sequential days.

Standard withanolide-A and withaferin-A were eluted at a retention time of 3.3 and 5.3 minutes, respectively. In this method, the limit of detection (LOD) was found to be 0.23 and 0.26 μg/ml, respectively.

Although, all the samples tested were polyherbal and had a label claim for the presence of Ashwagandha, only three samples (A, B, and D) had claim for the percentage of Ashwagandha. In addition, only sample B had identified the marker compound (withanolides) and mentioned its adjusted percentage. Further, the labels failed to clarify the amount of withanolides, dosage, indications or storage conditions.

**Figure 1:**
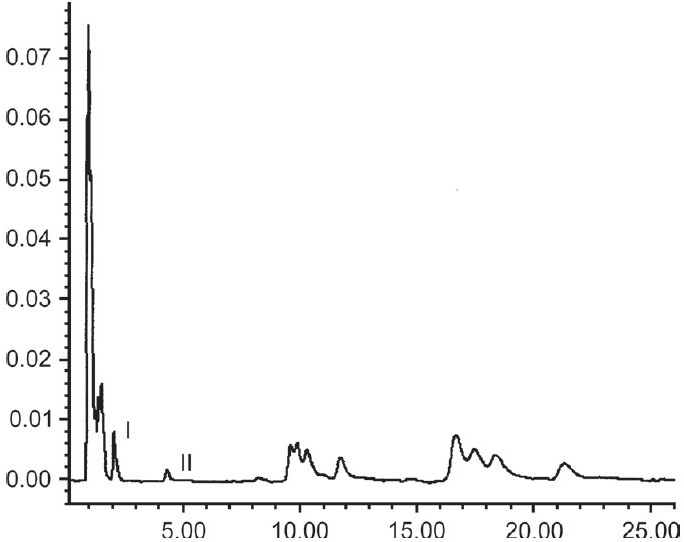
RP-HPLC of extracted sample B, with base separated peaks of withanolide-A (I) and withaferin-A (II)

**Figure 2:**
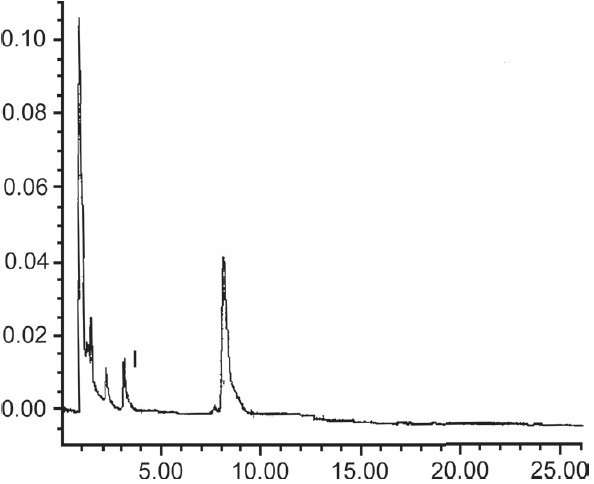
RP-HPLC of extracted sample D, with base separated peak of withanolide-A (I)

The developed HPLC method gave optimum separation of the constituents of the methanolic extract of the different samples. Under these conditions, base separated peaks of withanolide-A and withaferin-A could be identified and quantified. Chromatogram of the sample B clearly showed base separated peaks of withanolide-A and withaferin-A [Figure 1]. In contrast, the chromatogram of sample D revealed the presence of withanolide-A only [Figure 2]. Using the external calibration method, the concentrations of withanolide-A and withaferin-A were calculated in each sample [Table 1]. The results revealed a wide variation in the quantity of the two chemical constituents present in the products that are available in the market. In sample D, the quantity of withaferin-A could not be assessed as it was below the LOD.

**Table 1:** Concentrations of withanolide-A and withaferin-A in different formulations

*Sample*	*Withanoloid-A*	*Withaferin-A*
A	32.17 μg/tab	2.8 μg/tab
B	14.03 μg/tab	1.5 μg/tab
C	574.5 μg/g powder	1.75 μg/g powder
D	14.4 μg/ml syrup	Not detectable

This study highlights the gross liberties enjoyed by the manufacturers of medicinal plant-based products in India. The label claims are inadequate regarding the contents, their quantity, and quality or usage/storage advice of the products. In light of these inconsistencies, the user is being misled regarding the benefit of these products.

Bulk manufacturing of standardized plant-based products is challenging,[2] and in view of this, the United States Congress, passed the Dietary Supplement Health and Education Act.[3,4] However, this is self-limiting as labeling requirements for dietary supplements are not as stringent as that for pharmaceuticals, and concerns regarding their purity and potency are not reduced. In India and in other developing countries, there is paucity of stringent and effective regulatory control on quality standards for sale of over-the-counter medicinal plant-based products, especially regarding drugs' chemical instability, inappropriate storage, and transport, etc. Under the present scenario, the naïve masses are at the compromising end regarding the safety and efficacy of these “health products.” The issue of inconsistency in the chemical constituents of the medicinal plant-based products is a dynamite waiting to explode and needs immediate redress.
